# Two-Component FAD-Dependent Monooxygenases: Current Knowledge and Biotechnological Opportunities

**DOI:** 10.3390/biology7030042

**Published:** 2018-08-02

**Authors:** Thomas Heine, Willem J. H. van Berkel, George Gassner, Karl-Heinz van Pée, Dirk Tischler

**Affiliations:** 1 Institute of Biosciences, Environmental Microbiology, TU Bergakademie Freiberg, Leipziger Str. 29, 09599 Freiberg, Germany; heinet@tu-freiberg.de; 2 Laboratory of Biochemistry, Wageningen University & Research, Stippeneng 4, 6708 WE Wageningen, The Netherlands; willem.vanberkel@wur.nl; 3 Department of Chemistry and Biochemistry, San Francisco State University, 1600 Holloway Avenue, San Francisco, CA 94132, USA; gassner@sfsu.edu; 4 Allgemeine Biochemie, Technische Universität Dresden, 01062 Dresden, Germany; karl-heinz.van_pee@tu-dresden.de; 5 Microbial Biotechnology, Ruhr University Bochum, Universitätsstr. 150, 44780 Bochum, Germany

**Keywords:** hydroxylation, epoxidation, halogenation, heteroatom oxidation, biocatalysis, flavoprotein monooxygenases, flavin reductase

## Abstract

Flavoprotein monooxygenases create valuable compounds that are of high interest for the chemical, pharmaceutical, and agrochemical industries, among others. Monooxygenases that use flavin as cofactor are either single- or two-component systems. Here we summarize the current knowledge about two-component flavin adenine dinucleotide (FAD)-dependent monooxygenases and describe their biotechnological relevance. Two-component FAD-dependent monooxygenases catalyze hydroxylation, epoxidation, and halogenation reactions and are physiologically involved in amino acid metabolism, mineralization of aromatic compounds, and biosynthesis of secondary metabolites. The monooxygenase component of these enzymes is strictly dependent on reduced FAD, which is supplied by the reductase component. More and more representatives of two-component FAD-dependent monooxygenases have been discovered and characterized in recent years, which has resulted in the identification of novel physiological roles, functional properties, and a variety of biocatalytic opportunities.

## 1. Introduction

Enzymatic reactions are often highly chemo-, regio- and enantioselective and are therefore of high interest for biotechnological applications. However, many enzymes require a certain cofactor to realize a catalytic reaction. These cofactors can be divided into prosthetic groups and coenzymes/co-substrates. Cofactors can be inorganic (metal ions), organic (e.g., flavin, pterin), or organometallic (e.g., heme, cobalamin). A huge set of organic cofactors are employed by enzymes, which can be vitamins and derivatives, sugars, small peptides and lipids (e.g., adenosine triphosphate (ATP), nicotinamide adenine dinucleotide (phosphate) (NAD(P)), coenzyme A, pyridoxal phosphate, flavin adenine dinucleotide (FAD), flavin mononucleotide (FMN), vitamin K, glutathione). Cofactors allow the transfer of chemical groups as well as electron transfer in redox reactions. Further, they can build together with the protein a three-dimensional space and thus form the catalytic active site, fostering substrate binding and/or conversion.

**Figure 1:**
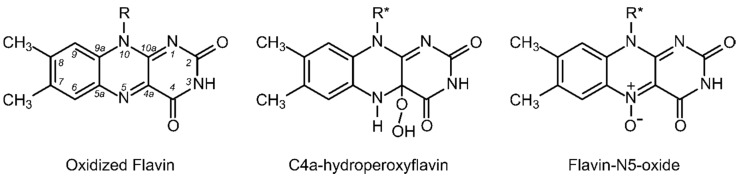
(**left**) Chemical structure of the oxidized form of the flavin isoalloxazine ring system with indication of the atom numbering. (**middle**) Chemical structure of the C4a-hydroperoxyflavin. (**right**) Chemical structure of the flavin-N5-oxide. R = ribityl (Riboflavin), ribitylphosphate (flavin mononucleotide, FMN) or ribityl-ADP (flavin adenine dinucleotide, FAD); R* = ribitylphosphate or ribityl-ADP.

Amongst the most versatile cofactors are the riboflavin (vitamin B2) derivatives, flavin adenine dinucleotide (FAD) and flavin mononucleotide (FMN) [1]. They can occur in flavoproteins in covalently bound (~10%) as well as dissociable forms [2,3]. They are able to perform one- and two-electron transfer reactions, which enable them to support a wide range of catalytic reactions and biological processes [4,5]. The catalytically important moiety is the isoalloxazine ring (Figure 1), which can adopt several redox states [6]. 

One specific group of flavoproteins that has received increasing attention in biotechnology concerns the flavin-dependent monooxygenases. These enzymes act on many different substrates by utilization and activation of molecular oxygen via the reduced form of the flavin cofactor (Figure 1) [7,8,9,10,11]. Flavoprotein monooxygenases (EC 1.14.13, EC 1.14.14, EC 1.13.12) are widespread in nature and can be classified in several groups according to their fold and function [7,8]:

Single component monooxygenases, belonging to groups A and B, contain a tightly bound FAD cofactor and are dependent on NAD(P)H as the external electron donor. A notable exception is the recently discovered 2-aminobenzoylacetate *N*-hydroxylase (PqsL) from *Pseudomonas aeruginosa*. PqsL contains a FAD cofactor but depends on free reduced flavin as external electron donor instead of NAD(P)H [12]. Single-component Baeyer-Villiger monooxygenases (group B) are especially useful for biocatalytic applications, because they catalyze a wide range of reactions with high enantioselectivity [10,13,14,15,16].

Two-component flavoprotein monooxygenases use either reduced FMN (group C) or reduced FAD (groups D, E and F) for molecular oxygen activation. The reduced flavin that binds in the monooxygenase active site is delivered by a NAD(P)H-dependent flavin reductase [8,17]. Both protein components are usually encoded next to each other on the genome in a respective gene cluster. Direct interaction between monooxygenase and reductase is often not required and supply of reduced flavin can be as well compensated by other reductases, for instance in whole cell systems. 

Further, in single component internal flavoprotein monooxygenase (groups G and H), flavin reduction can be accomplished via the substrate [7,10,18].

During the enzymatic activation of molecular oxygen, a C4a-(hydro)peroxyflavin is formed (Figure 1), which can act as nucleophile or electrophile [11,19]. Subsequently, one oxygen atom is inserted into the substrate while the other one is reduced to water. Insertion of oxygen into the substrate is in many cases chemo-, regio- and enantioselective [20,21,22]. Stabilization of the highly reactive C4a-(hydro)peroxyflavin intermediate within the enzyme active site is critical for the monooxygenase activity and directs the mode of the catalytic reaction [23].

Some recently discovered flavoprotein monooxygenases utilize a flavin-N5-oxide for monooxygenation (Figure 1). So far, only three of them have been characterized [24,25,26]. Their unique properties might allow for additional applications of flavoprotein monooxygenases [27].

Cofactor regeneration is one of the biggest issues concerning flavoprotein monooxygenases and their industrial application [28]. Especially, application of two-component variants can become complex and cost intensive. Besides regeneration of the flavin co-substrate, additional components are required for recycling of NAD(P)H (e.g., by means of dehydrogenases) and for prevention of oxidative stress (catalases). Oxidative stress can occur when reduced flavin reacts with oxygen to form hydrogen peroxide, a process that for monooxygenases is often referred to as unproductive uncoupling [9,29].

Numerous efforts have already been made for the improvement of the biocatalytic properties of flavin-dependent monooxygenases. Whole cell systems have been used to accomplish supply of reduced flavin by cellular metabolism [30,31,32,33,34]. Further, enzymatic regeneration (e.g., by regeneration of the nicotinamide cofactor) was examined in cell-free systems and is particularly of interest in cascade systems and coupled reaction where biocatalysts complement each other [35]. In addition, it is possible to reduce the complexity of the systems to generate an economic and eco-friendly process. Therefore, flavins were regenerated non-enzymatically by applying organometallic catalysts, electrochemical approaches, and photochemical procedures [36,37,38]. A promising alternative is the supply of nicotinamide analogues for direct reduction of flavins [39,40,41,42]. These cheap biomimetics can be supplied in stoichiometric amounts and bypass a main hindrance towards the application of two-component flavin-dependent monooxygenases [43].

Here we put focus on the properties of two-component FAD-dependent monooxygenases. These enzymes are involved in the degradation of a variety of (xeno-)biotics that accumulate in the environment and which are often harmful. They also are key players in the biosynthesis of various secondary metabolites and therefore of huge interest for industrial applications. Their scope of catalyzed reactions offers the opportunity to introduce them into (bio-)chemical synthesis routes. In this review, special emphasis is given to the physiological role and functional properties of new members that were discovered in recent years, and to the biocatalytic opportunities and optimization of certain family members. Newly discovered biochemical and structural features of the monooxygenase and reductase components of specific members are explained as well. Information about the biochemical properties and biotechnological applications of two-component FMN-dependent monooxygenases (group C) can be found in other reviews [17,44].

## 2. Two-Component FAD-Dependent Monooxygenase Systems

According to the recent classification [7], two-component FAD-dependent monooxygenases belong to groups D, E, and F. They structurally differ from the group C enzymes, which have a TIM-barrel fold. Groups E and F enzymes contain a Rossmann-type FAD-binding domain, while group D enzymes have a CATH domain, which can be found in acyl-CoA dehydrogenases [45]. Some group D monooxygenases can utilize FMN, while group E and group F members are strictly FAD-dependent [7,45].

Two-component FAD-dependent monooxygenases are involved in the catabolism and detoxification of aromatic compounds, amino acids, vitamins, cofactors, and in the biosynthesis of secondary metabolites by catalyzing hydroxylations, epoxidations, or halogenation reactions. Many of them are able to convert derivatives of their physiological substrate and some also perform heteroatom oxidations. This promiscuity is of interest for synthesis of fine chemicals, pharmaceuticals, agrochemicals, and makes these enzymes of value for biotechnological applications [21,22,28,32,46,47].

### 2.1. Group D Flavoprotein Monooxygenases

**Figure 2:**
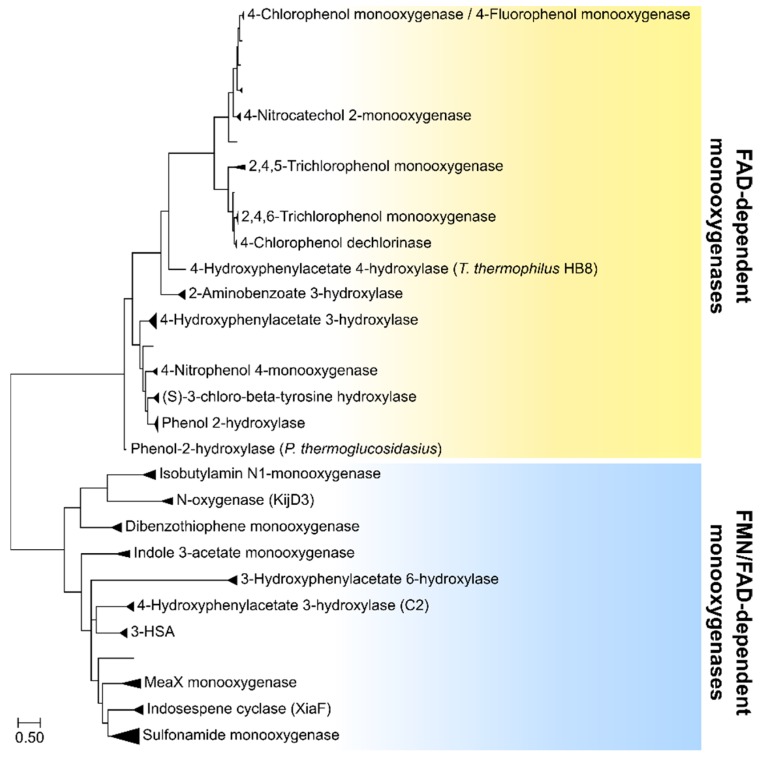
Condensed phylogenetic distance tree of group D monooxygenases. Group D enzymes can be distinguished into FAD and FAD/FMN-dependent clades. Amino acid sequences were aligned by using the ClustalW algorithm and after phylogenetic analysis, the maximum likelihood tree was constructed applying the MEGA7 software with bootstraps of 1000 replicates [50].

Group D flavoprotein monooxygenases (Table 1) catalyze hydroxylation of aromatic compounds but also *N*-hydroxylation reactions are described. Members of group D can be divided into FAD- and FMN-dependent enzymes, while some are also promiscuous towards the flavin co-substrate [7,48,49]. This clustering is also reflected by phylogenetic analysis of group D (Figure 2).

Two of the first characterized members are the phenol 2-hydroxylase and the 4-hydroxyphenylacetate monooxygenase, which will be described in the first place to outline a general overview about FAD-dependent group D monooxygenases. Many group D monooxygenases that originate from *Streptomyces* are involved in the biosynthesis of secondary metabolites (antibiotics, antitumor agents) [48,49,60,68]. For example, a recently discovered enzyme (XiaF) is involved in the biosynthesis of an indolosesquiterpene (xiamycin) [48]. Other recently characterized members are involved in the degradation and detoxification of halogenated phenols [58,59]. One of the latter ones, HadA, performs an oxidative dechlorination and will also be highlighted in the following section [59]. 

#### 2.1.1. Phenol 2-Hydroxylase

**Scheme 1:**
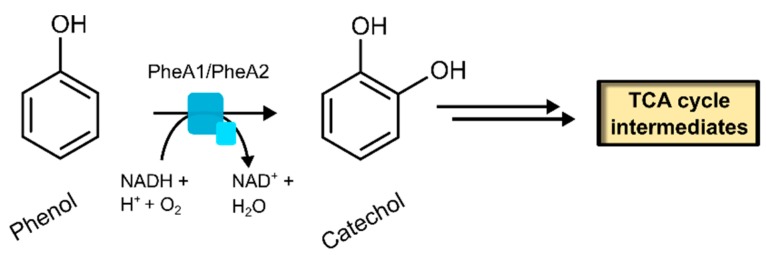
Hydroxylation at the *ortho*-position of phenol by PheA1. Reduced FAD is supplied by the second component PheA2. The product catechol is subsequently converted into TCA cycle intermediates.

**Physiological context**. Two-component phenol hydroxylases (PHs) consist of a monooxygenase (PheA1; EC: 1.14.14.20) and a flavin reductase (PheA2; EC: 1.5.1.-). They catalyze the first step of the degradation of phenol in several aerobic microorganisms. For that, phenol is hydroxylated at the *ortho*-position to catechol (Scheme 1) [55,69,70]. Catechol can be further catabolized via *ortho*- or *meta*-cleavage pathways into metabolites of the TCA-cycle [71]. PHs were identified and characterized in mesophilic organisms (*Rhodococcus*) but also thermophilic organisms (*Geobacillus thermoglucosidasius* A7; *Geobacillus stearothermophilus*) [70,72,73,74,75].

**Biochemistry**. The monooxygenase component PheA1 is a 44–57 kDa protein with a high amino acid (aa) sequence identity to other two-component aromatic hydroxylases like 4-hydroxyphenylacetate hydroxylase and 4-nitrophenol 2-hydroxylase. The FAD cofactor cannot be replaced by other flavins like FMN or riboflavin. PheA1 from *G. thermoglucosidasius* A7 is active as homodimer and thermostable up to 60 °C with an optimum at 55 °C [69]. Interestingly, PheA1 from *Rhodococcus erythropolis* UPV-1 exists as homotetramer [73].

PheA1 enzymes can further catalyze the hydroxylation of phenol derivatives like 2-methylphenol, 2-chlorophenol, 3-methylphenol, 3-nitrophenol, 4-methylphenol, 4-nitrophenol, 4-chlorophenol, 4-fluorophenol, 2,4-dichlorophenol, and resorcinol but not benzoate, orcinol, 4-hydroxybenzoate, 4-hydroxyacetophenone, and 4-hydroxyphenylacetate [69,70,73,74]. Not all substrates were assayed for all enzymes; see respective references for detailed information.

The reductase component PheA2 is a 16–19 kDa protein with about 30% aa sequence identity to the styrene monooxygenase reductase StyB from *Pseudomonas fluorescens* [69,70,73]. PheA2 is a NADH-dependent reductase and contains FAD as a prosthetic group. A tightly bound FAD (*K*_d_ ~ 10 nM) is rather uncommon for such short-chain reductases [76]. Thus, a second FAD molecule (*K*_M_ ~ 1.5 μM) is used as substrate to establish a ping pong-bi-bi-mechanism [69]. PheA2 from *G. thermoglucosidasius* A7 is active as homodimer and thermostable up to 65 °C (>65% of initial activity) with an optimum of 55 °C. Phenol did not influence the rate of NADH oxidation or oxygen consumption [69]. PheA2 has a clear preference for NADH over NADPH and catalyzes the reduction of FAD with a *k*_cat_ of about 250 s^−1^. FMN and riboflavin are reduced at similar rates but the affinity for these flavins is 3–4 times lower than for FAD [69].

PheA2 from *R. erythropolis* UPV-1 contains, in contrast to the previous variant, no tightly bound flavin and follows a random sequential mechanism [73]. Besides being less thermostable, it shows lower conversion rates and a lower affinity for the FAD substrate than PheA2 from *G. thermoglucosidasius* A7. Both PheA2 reductases can utilize NADPH as electron donor, although with a lower efficiency compared to NADH. No physical interaction between monooxygenase and reductase is necessary for transfer of reduced FAD [69,73].

**Figure 3:**
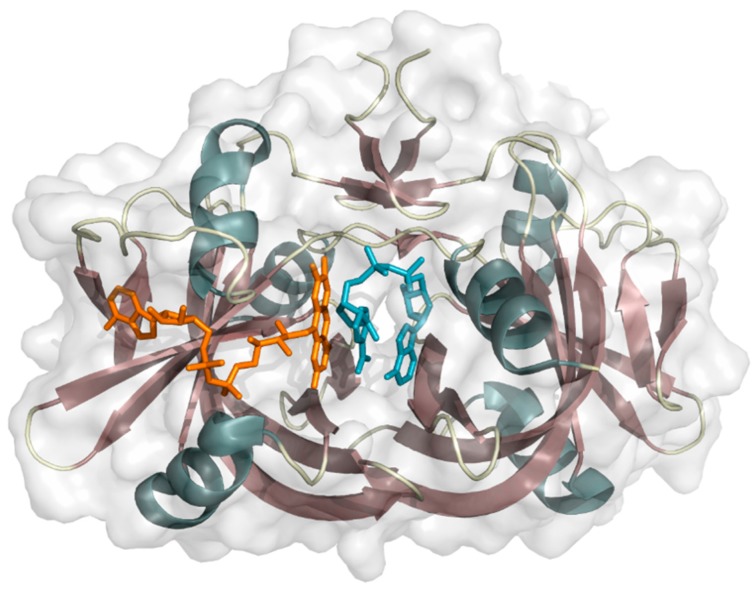
Structure of PheA2 dimer (Protein Data Bank (PDB) ID: 1RZ1). Coloring is according to secondary structure: helices (deep teal), sheets (brown) and loops (pale yellow). Surface is indicated transparently. For clarity, only one FAD (orange) and nicotinamide adenine dinucleotide (NAD) (light blue) is illustrated per dimer. Note that the FAD is reduced and that the NAD coenzyme is bound in a stacked conformation.

**Structure**. The biochemical differences between the thermophilic-like PHs and mesophilic-like PHs are also reflected by phylogenetic analysis [74]. This is probably due to the adaption of the bacterial host to the habitat conditions. However, so far, only the crystal structure of the reductase component PheA2 from *G. thermoglucosidasius* A7 is available (Protein Data Bank (PDB) ID: 1RZ1; Figure 3) [76]. PheA2 is structurally related to ferric reductase (FeR), although no such activity was reported [69,76].

The core of PheA2 is formed by a six-stranded antiparallel *β*-barrel, which is capped by an *α*-helix. PheA2 lacks a Rossmann fold domain, but is able to bind the nicotinamide coenzyme in a stacked mode in the cleft next to the isoalloxazine ring of FAD (Figure 3). As already mentioned, PheA2 contains a tightly bound FAD as a prosthetic group. Most other related flavin reductases weakly bind FAD as co-substrate. The strong affinity of PheA2 for FAD might arise from a loop located at the protein surface, which embeds the adenine moiety of FAD. PheA2 does not tightly interact with FMN, suggesting that the adenine moiety is essential for binding as prosthetic group [76]. The isoalloxazine ring of the FAD prosthetic group and the nicotinamide ring of the pyridine nucleotide co-substrate are located at the dimer interface of PheA2. The double-stacked conformation of NAD with reduced FAD (Figure 3) is rather unusual but might be necessary to stabilize the interaction between the cofactors in the solvent exposed active site. After reduction of the FAD, the NAD^+^ is released and a substrate FAD binds at the NAD binding site of PheA2 and might adapt a similar conformation. The affinity for the FAD substrate is much weaker than for the cofactor FAD [76]. PheA2 is still unique amongst this type of reductases, although recently an artificial homolog was constructed that might allow for further insights into the origin of the properties of PheA2 [77].

#### 2.1.2. 4-Hydroxyphenylacetate 3-Hydroxylase

**Scheme 2:**
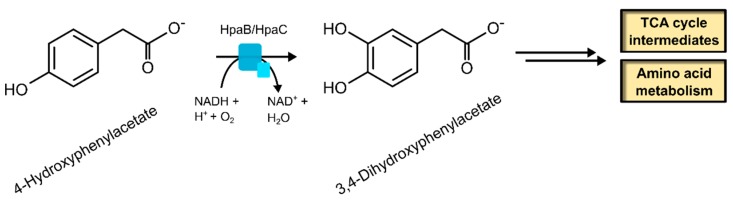
HpaB-mediated hydroxylation of 4-hydroxyphenylacetate to 3,4-dihydroxyphenylacetate. Reduced FAD is supplied by the second component HpaC. The product is subsequently converted into TCA cycle intermediates but is also part of the tyrosine metabolism.

**Physiological context**. Two-component 4-hydroxyphenylacetate 3-hydroxylases (HPAH; EC: 1.14.14.9) are involved in the catabolism of 4-hydroxyphenylacetate. Many microorganisms can use this aromatic compound as sole source of carbon and energy. To initiate its biodegradation, a second hydroxyl group is introduced by an inducible flavin-dependent hydroxylase (HPAH; Scheme 2). The product of HPAH, 3,4-dihydroxyphenylacetate can undergo *meta*-cleavage and is funneled into the TCA cycle [78,79]. 

Two different types of two-component 4-hydroxyphenylacetate 3-hydroxylases are known [7]. Although the overall fold of both monooxygenase components is similar, the one from *Acinetobacter baumannii* (C_2_) employs a large FMN-dependent reductase (35 kDa; C_1_) that contains a substrate-binding regulatory domain [80,81,82,83,84]. Interestingly, the monooxygenase component C_2_ is promiscuous towards the flavin co-substrate [82,83].

Furthermore, a FAD-dependent HPAH system is known (HpaB, oxygenase; HpaC, reductase), which shows higher similarities to most other two-component systems [54]. The FAD-dependent HPAH system was found in several organisms (*Arthrobacter* sp., *Escherichia coli*, *Geobacillus* sp., *Halomonas* sp., *Klebsiella pneumonia*, *Pseudomonas* sp., *Sulfolobus tokodaii*, *Thermus thermophilus*) and will be described here in detail.

**Biochemistry**. The HpaB monooxygenase component has a size of about 54–65 kDa and shares at least 28% amino acid sequence identity [85,86,87,88]. It is active as homodimer and able to bind and stabilize reduced FAD in absence of the substrate in order to prevent autooxidation of the co-substrate [54,85,89]. Oxygenation of 4-hydroxyphenylacetate is highly FAD-dependent [90].

The monooxygenase is able to act on several other aromatic compounds including (halo)phenols, (halo)catechols, cresols, amino acids, phenylpropanoids, and resveratrol among others [31,33,78,80,86,91,92]. Not all substrates were assayed for all enzymes; see respective references for detailed information. In some cases, NADH consumption was used to determine the activity on a substrate and therefore unproductive NADH oxidation instead of product formation cannot be excluded.

The reductase component has a size of 16–19 kDa with a weakly bound FAD as co-substrate [89]. It is likely that the reductase is active as homodimer, although studies also found the monomeric form in solution [85,89,90]. The reductase prefers NADH over NADPH as substrate. It binds FAD with an affinity in the low µM range and does not discriminate between other flavins [89,90].

In contrast to the FMN-dependent system, the HpaC reductase activity is not allosterically regulated by 4-hydroxyphenylacetate and the HpaB monooxygenase component does not directly interact with the reductase component [54,89,90,93]. However, it cannot be excluded that some protein–protein interaction influences the overall reaction [89,90]. Effective coupling is dependent on the molar ratio of the monooxygenase and reductase component [80,85,89].

**Structure**. The crystal structure was determined for the FMN-dependent monooxygenase from *Acinetobacter baumannii* (PDB ID: 2JBT) and for the FAD-dependent system from *Thermus thermophilus* HB8 (PDB ID: 2YYJ). The overall folding of both HPAHs is very similar but there are significant deviations at the cofactor and substrate-binding site. For instance, the adenine moiety of FAD binds at a loop that is not present in the *Acinetobacter* enzyme, which is in accordance with the FMN-dependent nature of monooxygenase C2 [89]. 

**Figure 4:**
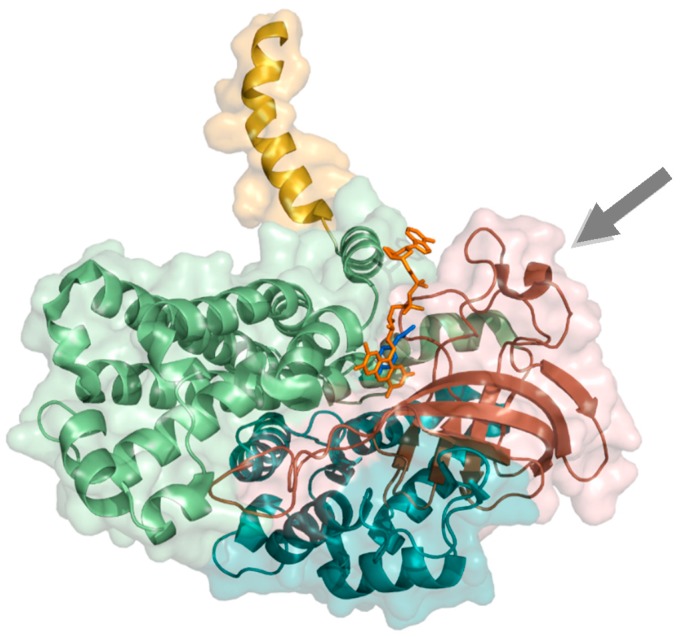
Structure of 4-hydroxyphenylacetate 3-hydroxylase (HPAH) monomer (PDB ID: 2YYJ). Coloring is according to the *N*-terminal domain (teal), middle domain (brown), the *C*-terminal domain (pale green), and the *C*-terminal tail (yellow). The surface is indicated transparently, FAD (orange) and 4-hydroxyphenylacetate (blue). The loop region that allows recognition of the adenine moiety of FAD is indicated by an arrow.

The monomer of HpaB consists of three domains and a *C*-terminal tail [94]. The *N-*terminal and *C*-terminal domain contain mainly antiparallel *α*-helices, while the middle domain contains *β*-sheets forming a barrel-like structure (Figure 4). A cave at the interspace of the three domains allows binding of reduced FAD and the substrate. FAD adopts an extended conformation, while also residues of the adjacent monomer are required for binding. A conformational change was observed in a loop region of the middle domain upon binding of FAD, which is hypothesized to be responsible for the preference of FAD over FMN as co-substrate. The cavity is largely open to the solvent and allows access of the substrate and oxygen after binding of the co-substrate. An additional movement of another loop in the middle domain shields the active site from the solvent after binding of the substrate. The substrate is located at the *re* face of the isoalloxazine ring [94].

The hydroxyl group of 4-hydroxyphenylacetate is oriented by conserved residues, which interact in other enzymes with analogous compounds. In contrast, the residues that direct the carboxyl group of the substrate are not conserved. The aromatic moiety is layered by hydrophobic residues [94]. As mentioned previously, the C4a-hydroperoxyflavin is stabilized in the active site as long as no substrate is present. This is assumed to be achieved through hydrogen bonding with an arginine, which is conserved in similar monooxygenases, and further, a threonine and the N5 atom of the flavin isoalloxazine ring [89,94,95].

The structure of reductase HpaC from *T. thermophilus* HB8 (PDB ID: 2ED4, *T. thermophilus* HB8; 2D37, *Sulfolobus tokodaii* 7) resembles that of PheA2 (cf. Figure 3) [96,97]. Comparison of apo- and holoenzymes showed only minor conformational differences between both states [97]. Soaking experiments of HpaC with NADPH revealed binding in a stacked mode similar to the binding mode of NADH. However, NADPH was positioned upside down in the active site, unfavorable for hydrogen transfer [96]. This might explain why these FeR-like reductases are strictly NADH-dependent. The position of the FMN moiety and NADH is almost identical to that of PheA2 and related reductases. Interestingly, differences were found in a loop, which is hypothesized to interact with the AMP part of FAD. This loop is less conserved in length and amino acid sequence [97]. As HpaC, in contrast to PheA2, contains a weakly bound FAD, it is likely that this loop defines whether FAD can also function as a prosthetic group [97].

#### 2.1.3. 4-Chlorophenol Dechlorinase

**Physiological context**. The FAD-dependent monooxygenase HadA catalyzes the dechlorination of (poly)-chlorinated phenols with a preference for *para*-substituted substrates [59]. Such chlorinated compounds are often used as pesticides in agricultural industries and are therefore often contaminants in the environment. While being formerly denoted as persistent, several microorganisms are able to degrade these compounds. The required replacement of the chloride is often catalyzed by group D hydroxylases [56,57,98]. The dechlorinase HadA originates from *Ralstonia eutropha* JMP134 and displays a broader substrate spectrum than homologous enzymes [59].

**Biochemistry**. A recent investigation of HadA has uncovered the first mechanistic study of oxidative dechlorination by two-component flavin-monooxygenases [59]. HadA is a 59-kDa protein highly similar to previously characterized trichlorophenol monooxygenases [57,98]. It can only bind and utilize FAD as a cofactor [59]. In single turnover reactions, HadA catalyzes the incorporation of a hydroxyl group at the *para*-position of the substrate. When this position contains a chlorine substituent, simultaneous dechlorination occurs [59]. Multiple chlorinated substrates are further hydroxylated at the remaining chlorinated positions of the phenolic ring until complete dehalogenation of the substrate occurs [59]. A detailed elucidation of the kinetic mechanism revealed that binding of reduced FAD to HadA leads to formation of two different protein•FADH^−^ species that react with O_2_ at different rates. Similar observations were already made, inter alia, for a group F flavoprotein monooxygenase (RebH) but not for other group D monooxygenases [59,99]. The observed reactivity of HadA with O_2_ was attributed to an intrinsic control mechanism for regulation of O_2_ diffusion into the active site or the formation of the C4a-hydroperoxyflavin [59].

The associated reductase component HadX has similarity to other NADH:flavin oxidoreductases like TcpX, TftC, and HpaC, but is so far not characterized in detail [100].

#### 2.1.4. Biotechnological Relevance of Group D Flavoprotein Monooxygenases

Hydroxylated aromatic compounds are of interest for chemical and pharmaceutical industries as they are used as precursor for chemical synthesis. This is in particular true for (halo)catechols, which can be produced by HPAHs and PHs [31,47,101]. HPAHs are able to produce cinnamic acid derivatives, which are potential antioxidants and anti-inflammatory agents [33,102]. Hydroxylated phenylpropanoids are of interest for pharmacological approaches [103,104].

Phenolic compounds are widely used in industrial processes and therefore may also be liberated into the environment. (Halo)phenolic substances are considered to be toxic and thus removal of them from wastewater and industrial sites is of high ecological relevance [46]. PHs can play a key role in this process as they initiate the degradation but were also found to be able to dechlorinate halophenols [59]. Further, PHs are of interest for synthesis of other hydroxylated compounds such as hydroxytyrosol [105].

Another promising feature is the activity of recently characterized group D flavoprotein monooxygenases with bulky compounds. Some hydroxylases are involved in the biosynthesis of the enediyne antitumor antibiotics like C-1027, kedarcidin, maduropeptin, and sporolides A and B [60,106,107,108]. Therefore, the substrate is tethered to a peptidyl carrier protein allowing for interaction with the monooxygenase [109]. This tethering can increase the efficiency of the catalysis and might therefore be a starting point for the optimization of the enzymes [110]. The requirement for a substrate that is bound to a carrier protein can also be found in group F monooxygenases (cf. Section 2.3.2).

**Figure 5:**
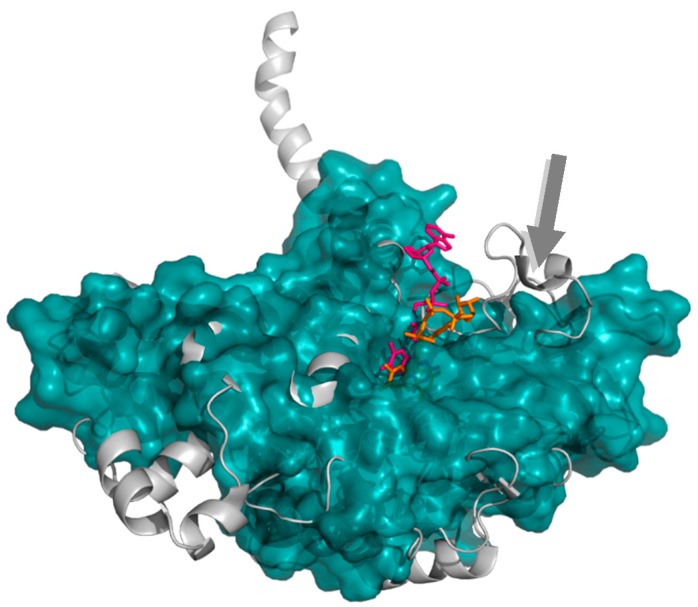
Structural comparison of the monomers of 4-hydroxyphenylacetate 3-hydroxylase (PDB ID: 2YYJ) and XiaF (PDB ID: 5MR6). HPAH is shaded in grey with bound FAD (purple). XiaF is displayed including surface (deep teal) with bound FAD (orange). The loop region and therefore the substrate-binding pocket (indicated by an arrow) is more open in XiaF, allowing for promiscuity towards the flavin co-substrate [48].

In addition, a better understanding about the flavin and substrate binding can facilitate enzyme design. As already mentioned, the loop region in the middle domain can influence flavin as well as substrate preference but potentially also the reactivity. For instance, the hydroxylase XiaF shows hydroxylation-induced terpenoid cyclization [48]. The active site of XiaF is more open what might help to accommodate bigger substrates and absence of the loop that is required for recognition of the adenine domain might explain the promiscuity towards the flavin co-substrate (Figure 5). A preference for FMN can also be found for other group D enzymes that are often involved in biosynthesis of antibiotics [68,111,112,113]. Swapping and changing of this region might allow conversion of bigger substrates but also other reactivities.

### 2.2. Group E Flavoprotein Monooxygenases

**Scheme 3:**
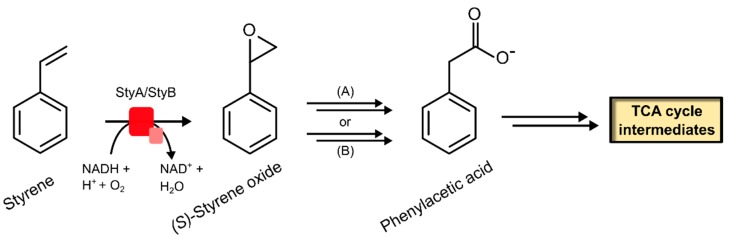
The upper styrene degradation pathway is initiated by the FAD-dependent epoxidation of styrene to (*S*)-styrene oxide. FAD is recycled by a NADH-dependent oxidoreductase StyB. StyA is usually part of a gene cluster that allows further degradation via a “classical” (A) or glutathione-dependent (B) pathway to the central intermediate phenylacetic acid, which is funneled via several steps into the TCA cycle.

**Physiological context**. To date, the only representatives that are assigned as group E flavoprotein monooxygenases are the styrene monooxygenases (SMOs) [7,8,22]. SMOs consist of a monooxygenase (StyA, SMOA; EC: 1.14.14.11) and a flavin reductase (StyB, SMOB; EC: 1.5.1.36). SMOs were described for the first time in *Pseudomonas* sp. [114] where they enable the degradation of styrene and its utilization as source of carbon and energy by catalyzing its conversion to (*S*)-styrene oxide (Scheme 3) [115]. The “classical” styrene degradation cluster consists of SMO, a styrene oxide isomerase (SOI) [116] and a phenylacetaldehyde dehydrogenase [117]. Since then, SMOs have been isolated and characterized from several other proteobacteria and actinobacteria [118,119,120,121]. These studies established that the organization of the gene cluster is heterogeneous, especially concerning the regulatory and transport machinery [120]. Recently, a gene cluster was discovered on a plasmid of *Gordonia rubripertincta* CWB2, where the SMO initiates an alternative styrene degradation pathway. Thereby, styrene oxide is converted by a glutathione *S*-transferase via additional steps to phenylacetic acid as a central intermediate [122].

**Figure 6:**
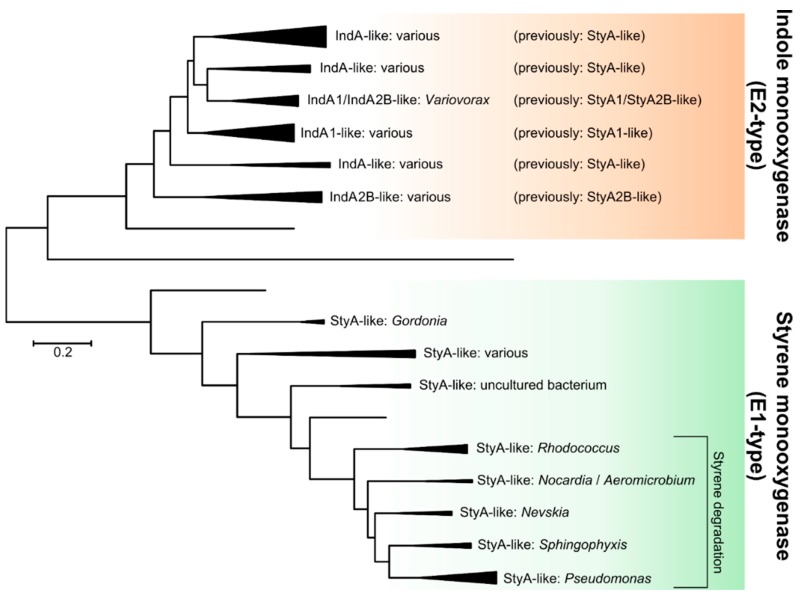
Condensed phylogenetic distance tree of group E monooxygenases. Group E enzymes cluster in two different types (E1 and E2). Styrene monooxygenases (SMOs) that are edged by a bracket are proven or likely involved in styrene degradation, as the strains harbor cognate gene clusters [118,119,120,121,122]. Amino acid sequences were aligned by using the ClustalW algorithm and after phylogenetic analysis, the maximum likelihood tree was constructed applying the MEGA7 software with bootstraps of 1000 replicates [50]. Fusion proteins were truncated for the reductase part.

However, the number of enzymes that are assigned to group E exceeds the number of enzymes that are located in one of these styrene degradation gene clusters by far (Figure 6). Moreover, a natural fusion of the monooxygenase and the reductase (StyA2B) was found some years ago in *Rhodococcus opacus* 1CP (*Ro*StyA2B) [123] and marks the beginning of the diversification into two subtypes E1 and E2 (Figure 6) [22]. The prototype StyA2B usually clusters together with a separate monooxygenase StyA1 [124]. The corresponding oxygenase components of the StyA1/StyA2B system share about 50% amino acid sequence identity [125]. Recently, such a cluster was found in the *β*-proteobacterium *Variovorax paradoxus* EPS and was characterized (*Vp*StyA1/*Vp*StyA2B) [125,126]. The *Variovorax*-like enzymes form a separate branch within the phylogenetic tree and there are indications for a convergent evolution of the StyA1/StyA2B-systems, also because the *Variovorax* variant possesses higher sequence identity (74%) between the monooxygenase subunits [125].

**Scheme 4:**
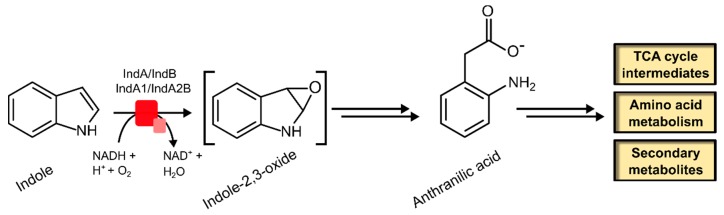
Indole degradation and detoxification is initiated by the FAD-dependent epoxidation performed by IndA or IndA1/IndA2B. FAD is recycled by a NADH-dependent oxidoreductase IndB or IndA2B. It is not clear yet whether the product of this reaction is an epoxide or indole-2,3-dihydrodiol. However, further degradation yields anthranilic acid, which can be used for the synthesis of secondary metabolites or amino acids.

Additionally, several branches of StyA-like enzymes emerge in E2-type monooxygenases (Figure 6). However, genomic and cluster analysis of the E2-type enzymes illustrate that there is no relationship of these enzymes to styrene degradation clusters. E2-type gene clusters often contain a short-chain dehydrogenase (SDR) and a dienelactone hydrolase (DLH; carboxymethylenebutenolidase) in proximity to the monooxygenase(s) [125]. Further, genes that are probably involved in degradation of aromatic (anthranilate, catechol, benzoate, phenol) and heteroaromatic compounds (indolepyruvate, xanthine) are encoded in these clusters. Recent studies suggest a role in indole metabolism and/or detoxification [125,127,128]. Thereby, the E2-type FAD-dependent monooxygenase initiates a catabolic cascade by epoxidation of indole. However, the product of indole oxygenation by those enzymes need to be described as there is no analytical proof of epoxidation, hydroxylation, or dioxygenation available, so far [128]. Usually indigo is formed under aerobic conditions by dimerization of indoxyl, which is prevented by the activity of the SDR-like enzyme and further converted into anthranilic acid by the DLH-like enzyme, acting as a cofactor-independent oxygenase (Scheme 4) [127,128].

According to the distinct physiological role and biochemical properties (see section below) of both subtypes, we herein introduce a reclassification of group E monooxygenases into “styrene monooxygenases” (SMOs; previously designated as E1-type) and “indole monooxygenases” (IMOs; previously designated as E2-type) (Figure 6, Table 2). IMOs catalyze the FAD-dependent epoxidation of indole. However, it is yet unclear if the product of the IMO is indole-2,3-oxide or indole-2,3-dihydrodiol, which is then rapidly transformed to indoxyl (Scheme 4) [128]. IMOs occur as two-component systems, which are composed of a FAD-dependent monooxygenase (IndA) and a reductase (IndB), and as self-sufficient fusion proteins (reassigned as ImoA2B) with an associated monooxygenase (reassigned as IndA1) [123].

#### 2.2.1. Styrene Monooxygenase

**Biochemistry**. The monooxygenase component StyA has a molecular mass of about 45 kDa. Many representatives from *Pseudomonas* spp. as well *Rhodococcus* spp. have been characterized in detail [121,129,130,131,132]. StyAs showed the ability to convert a variety of styrene derivatives as well as aryl alkyl sulfides in a regio- and enantioselective manner [43,121,129,132,133,134,135,136,137,138,139,140,141,142,143,144,145,146,147,148,149,150].

StyA contains a weakly bound FAD co-substrate and has a higher affinity for the reduced form of the flavin [129,130,151]. The inverse of this relationship exists for StyB, which has been shown to bind oxidized FAD more tightly than reduced FAD [152]. This arrangement facilitates thermodynamically the vectoral transport of reduced FAD from the reductase to the epoxidase and the return of oxidized FAD to the reductase [151]. StyA can operate in the absence of StyB provided a source of reduced FAD is available, but kinetic modeling suggests the most efficient coupling of NADH oxidation to styrene epoxidiation is consistent with the occurrence of a transient flavin-transfer complex involving the monooxygenase and reductase [130]. Single-turnover studies indicate that the oxygen half-reaction of StyA is not significantly influenced by StyB [131,152]. However, in catalysis by the StyA/StyB system, the catalytic activity of StyB is regulated by StyA. The transfer of FAD from StyB to StyA prevents the continuation of the StyB-catalyzed flavin reduction reaction during the course of the oxidative half-reaction of StyA and the half-life of StyB is greatly increased through its interaction with StyA during steady-state turnover [152]. On this basis, it is hypothesized that both components can form a transfer-state, which allows effective handover of the reduced FAD. The monooxygenase StyA forms homodimers in solution [121,129,151] and due to the sequestered position of the crystallographically-resolved dimer, it was postulated that this configuration represents the primary resting state of SMO [151]. These studies concerning the hydrodynamic state were carried out under oxidized conditions. Recently it was shown that StyA from *R. opacus* 1CP shifts to the monomeric form under reduced conditions, which shows that these monooxygenases can be active as monomers [121]. This state might be beneficial for protein-protein interaction with the reductase component StyB. 

The reductase component StyB has a molecular mass of about 18 kDa and is catalytically active as a dimer. StyB contains a weakly bound FAD as co-substrate and resembles the reductases that were mentioned in the previous sections [121,129,130,132,152,153]. The StyB reductase is rather unstable and tends to form inactive inclusion bodies upon expression. However, these inclusion bodies can be easily purified and refolded in the presence of FAD [129,132]. StyB is NADH-specific, but does not discriminate between FMN, FAD or riboflavin. StyB reductases follow an ordered sequential mechanism with NADH as leading substrate [129,130]. The active form of StyB is a homodimer and it was shown that the activity increases significantly upon dilution [121,129,130,132,152,153]. This is in accordance with the formation of inactive quaternary structures, especially at high concentrations. Therewith, turnover rates of over 500 s^−1^ are possible for reductases from *Pseudomonas* sp. [77,153].

**Figure 7:**
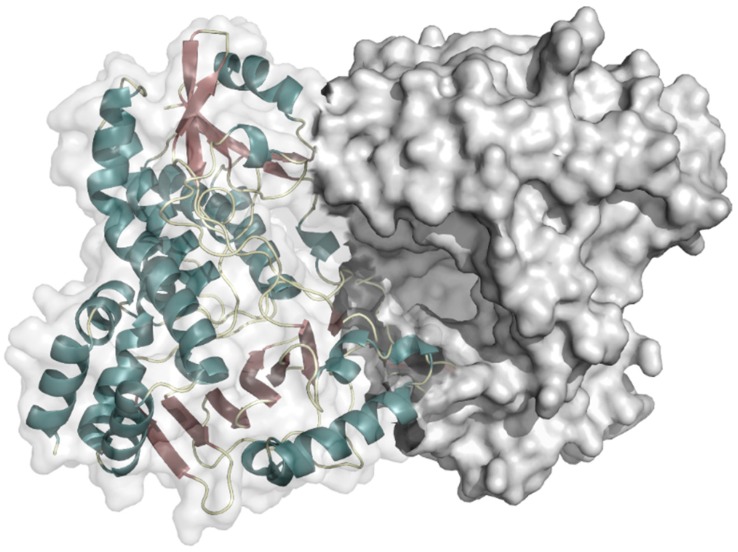
Structure of head-to-tail styrene monooxygenase dimer (PDB ID: 3IHM). Coloring is according to secondary structure: helices (deep teal), sheets (brown) and loops (pale yellow). The surface of the left monomer is indicated transparently [151].

**Figure 8:**
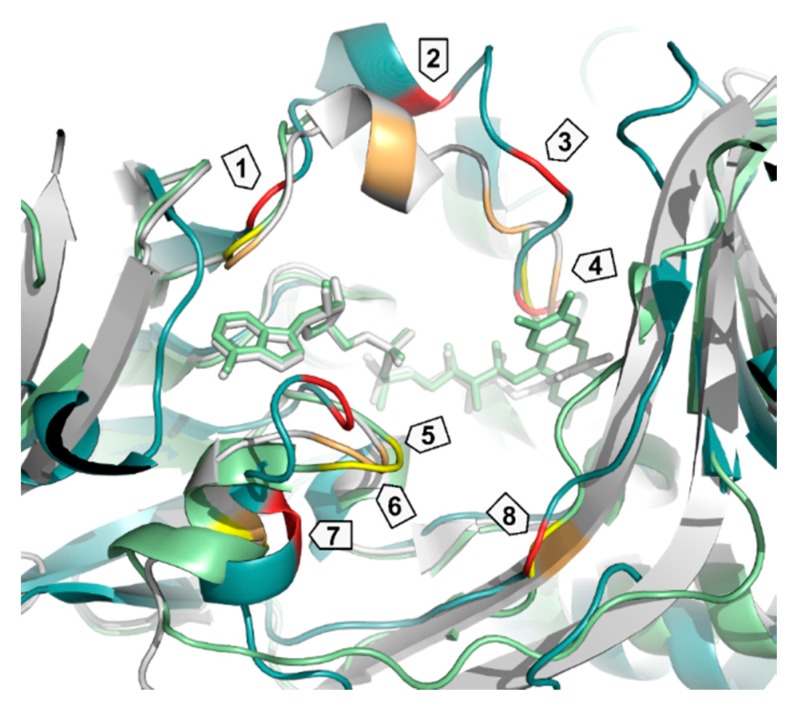
Comparison of the cofactor-binding site of *p*-hydroxybenzoate hydroxylase (PHBH) with 2-aminobenzoylacetate *N*-hydroxylase (PqsL) and SMOA. The crystal structures of SMOA (light teal) is compared with the group A enzymes PqsL (PDB ID: 2X3N, pale green) [12] and PHBH (PDB ID: 1PBE, grey) [155]. Critical residues for nicotinamide binding in PHBH are indicated (light orange) as well as the respective residues in SMOA (red) and PqsL (yellow). Numbering is in accordance with Table 3. The FAD ligands are shaded like the respective protein backbone.

**Structure**. The crystal structure of the monooxygenase component from *Pseudomonas putida* S12 (PDB ID: 3IHM) was solved by Ukaegbu et al., 2010 (Figure 7) [151]. SMOA forms a homodimer and the structure is related to group A *p*-hydroxybenzoate hydroxylase (PHBH). PHBH is a one-component system, which is able to reduce the flavin by itself. Therefore, a NAD(P)H binding site is needed [154], which is not present in SMOA [151]. Thus, SMOA seems to have lost or never had the ability for reduction of FAD and requires the external reductase StyB. This hypothesis is supported by investigations of 2-aminobenzoylacetate *N*-hydroxylase (PqsL), which is also not able to bind NAD(P)H [12]. In a similar manner, critical residues for recognition and binding of NAD(P)H are different to the group A prototype PHBH (Table 1) or adopt a considerably different orientation (Figure 8) [12,151].

It was not possible to obtain crystals of SMOA with bound FAD in oxidized or reduced form and/or substrate and hence the binding sites has been estimated by structural comparison to PHBHs (PDB ID: 1PBE). The putative substrate binding site is accessible via a cleft at the dimer interface of SMOA (Figure 7). This cleft accommodates one FAD molecule per monomer and enables entrance to the substrate cavity through a small tunnel, which is located beyond the isoalloxazine ring of FAD [151]. The hydrophobic cavity is formed between two loops above the seven stranded *β*-sheet. However, it is likely that the protein-FAD-substrate interaction is more complex as can be deduced from the apoprotein structure. PHBH contains a mobile flavin cofactor, which attains several different orientations during the catalytic cycle [156]. As the substrate-binding site in SMOA is not accessible for the isoalloxazine ring, it is likely that flavin and protein dynamics are needed to allow reaction between the oxygenated FAD and styrene [151].

The reductase component SMOB (or StyB) is homologous to group D-like reductases. This is reflected by high structural similarities between these enzymes [152]. However, the structure of StyB from *Pseudomonas putida* S12 (PDB ID: 4F07) is special in several points. It was shown that SMOB, refolded from inclusion bodies, is stabilized by the addition of FAD. Interestingly, the crystal structure was solved with each monomer binding two FAD molecules. The isoalloxazine ring of one FAD molecule is located at the active site of one monomer, while the adenine moiety of the same FAD is located in the nicotinamide binding pocket of an adjacent monomer [152]. Distinct differences at the loop region that were found to be responsible for interaction with the AMP part of FAD in related reductases are also observed in SMOB (cf. Discussion). This unusual binding mode of the FAD supports a possible interaction between the monooxygenase and the reductase component for the purpose of reduced FAD transfer as mentioned in the section before. Unfortunately, the *N*-terminal part of the reductase is not visible in the crystal structure. This would be of interest as recent studies show that this region influences the biocatalytic properties of these reductases [77]. Moreover, it was observed that NSMOB, which has an *N*-terminal extension of about 20 amino acids follows exclusively a double displacement steady-state mechanism and that the linker proteins which were observed follow a sequential ternary mechanism at low FAD concentration, but switch to a double displacement mechanism at higher concentrations of FAD [157].

#### 2.2.2. Indole Monooxygenase

**Biochemistry**. As already mentioned, IMOs can be discriminated into IndA/IndB- and IndA1/IndA2B-systems. With regard to our reclassification, so far 7 IMOs were shown to be active [124,126,127,158,159]. To date, IndA-like monooxygenase activity is solely attributed to indigo formation upon gene expression and therefore no additional information can be given with regard to biochemical properties [127,128,158,159]. However, detailed characterizations were conducted for IndA1/IndA2B-systems from *Rhodococcus* [121,123,124] and *Variovorax* [126] as well as for the reductase *Ab*IndB from *Acinetobacter baylyi* ADP1 [160].

IndA1s and IndA2Bs have a comparable activity as StyA-like enzymes for the conversion of styrene. Further, the substrate specificities are similar. However, the fusion proteins possess a much lower epoxidase activity than the associated IndA1s [124,126]. Therefore, it is hypothesized that IndA2B acts mainly as a reductase that provides reduced FAD for IndA2 and IndA1 [115]. The low epoxidation activity can be explained by the fact that *Ro*IndA2B stabilizes the FAD C4a-hydroxide upon styrene epoxidation, which supports the hypothesis that it is not the natural substrate for these enzymes [161]. Further, the low reductase activity of *Ro*IndA2B is assumed to result from the *N*-terminal fusion to the monooxygenase [77]. The phylogenetic distance between the *Rhodococcus* and the *Variovorax* variants (cf. Figure 6) is reflected in the biochemical properties. In general, the *Variovorax* enzymes have a higher activity and the reductive power of *Vp*IndA2B cannot be improved by an additional reductase, in contrast to *Ro*IndA2B [123,124,126].

As SMOs, IMOs are able to perform enantioselective epoxidations and sulfoxidations on a variety of (substituted) styrene derivatives and aryl alkyl sulfides [43,123,124]. In contrast to *Ro*StyAs, *Ro*IndA1 and *Ro*IndA2B showed a higher tendency to form oligomers and further the active form seems to be a dimer [121]. Although no direct interaction between the monooxygenase and the reductase is necessary [43] it was shown that the epoxidation activity of *Ro*IndA1 decreased, when *Ro*IndA2B is replaced by another reductase [17,124].

So far, only one IndB reductase from *A. baylyi* ADP1 has been characterized [160]. *Ab*IndB follows a random sequential mechanism in contrast to StyBs that display an ordered binding [129,130]. Above that, both reductase types are very similar.

**Structure**. There is no crystal structure of IMOs available yet. However, it is likely that the general fold of these enzymes is very similar to that of SMOs.

#### 2.2.3. Biotechnological Relevance of Group E Flavoprotein Monooxygenases

**Scheme 5:**
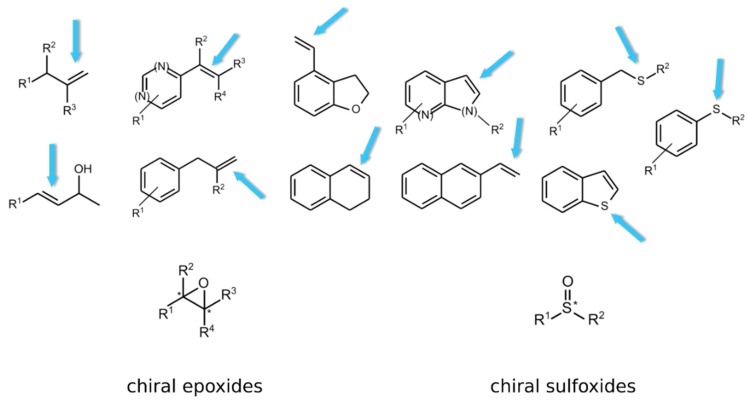
Illustration of substrates and products of group E flavoprotein monooxygenases [43,121,123,124,126,129,132,133,134,135,136,137,138,139,140,141,142,143,144,145,146,147,148,149,150,172]. Not all substrates were assayed for all enzymes; see respective references for detailed information.

In recent years, group E monooxygenases have gained a lot of attention in the field of oxidative biocatalysis [28,32,162,163,164,165,166,167]. SMOs and IMOs can catalyze the conversion of a huge variety of substrates (Scheme 5) with often exquisite enantioselectivity. While epoxidation is limited to the production of the (*S*)-enantiomer, several reports describe the generation of either (*S*)- or (*R*)-sulfoxides, depending on the biocatalyst and the substrate [124,126,138,145]. However, there is still space for optimization as enantiopurity of (*R*)-sulfoxides produced by group E monooxygenases is often not very high. 

Enantiopure epoxides are of relevance as building block for synthetic chemistry [168] and sulfoxides are used for instance in pharmaceutical and agricultural industries [165,169]. In addition, group E flavoprotein monooxygenases are able to produce indigoid dyes, as far as known, without the appearance of byproducts such as indirubin [170,171,172]. Indigoid dyes are of relevance for industrial and pharmaceutical applications [173,174,175,176,177,178,179]. 

Therefore, alternative cofactor regeneration systems have been developed for group E monooxygenases, including the direct regeneration by an organometallic catalyst, electrochemical approaches and the use of cofactor mimics [135,180]. Usability in a biocatalytic process has already been shown in liquid two-phase systems [181,182,183] and was transferred into large and pilot scale [30,184]. Further, group E monooxygenases can be used as modules in multi-enzymatic cascade reactions [150,168,185,186,187].

Beyond that, several studies showed that engineering of group E flavoprotein monooxygenases could be advantageous. It was possible to improve activity, enlarge substrate specificity and change enantioselectivity [32,77,139,141,143]. Generation of artificial fusions between the two-components monooxygenase and reductase showed that the linker has a huge impact on the biocatalytic mechanism and activity. It was therewith possible to increase efficiency of the chimeric enzyme [157,188].

### 2.3. Group F Flavoprotein Monooxygenases

Group F comprises a variety of flavin-dependent halogenases. These enzymes are involved in the production of secondary metabolites like antibiotics and antitumor agents and catalyze the regioselective halogenation of mostly aromatic substrates [189,190]. Group F can be distinguished into free-substrate halogenases and carrier protein-bound substrate halogenases, which also form separate clades in a phylogenetic tree (Table 4) [191,192]. An increasing interest in these halogenating enzymes is underlined by several reviews in recent years [191,193,194,195,196]. Here, a general overview of flavoprotein halogenases is presented and novel biotechnological aspects are outlined.

#### 2.3.1. Free Substrate Halogenases

**Scheme 6:**
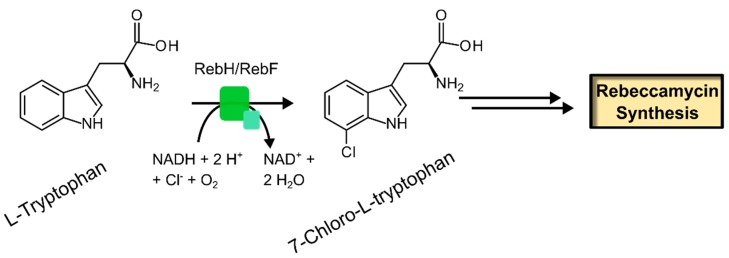
Chlorination of L-tryptophan by the halogenase RebH. FAD is recycled by the NADH-dependent oxidoreductase RebF. This reaction represents the initial step in the synthesis of the antitumor agent rebeccamycin [217].

**Physiological context**. Tryptophan halogenases were the first characterized representatives of group F and have been extensively studied. Tryptophan can be halogenated by these enzymes at the 2-, 4-, 5-, 6-, and 7-position of the indole ring [198,216]. The tryptophan 7-halogenase is so far the only representative of class F that has been assigned to EC classification (1.14.19.9). It is among others involved in the biosynthesis of the antitumor agent rebeccamycin, which is produced by *Lechevalieria aerocolonigenes* [217] (Scheme 6).

In addition, a recently characterized halogenase BvrH was shown to catalyze the bromination of indole at the C3 position and is not able to convert tryptophan [192]. In bacterial and fungal halogenases, halogenation can occur at different stages of biosynthesis of secondary metabolites [206,207,208].

**Figure 9:**
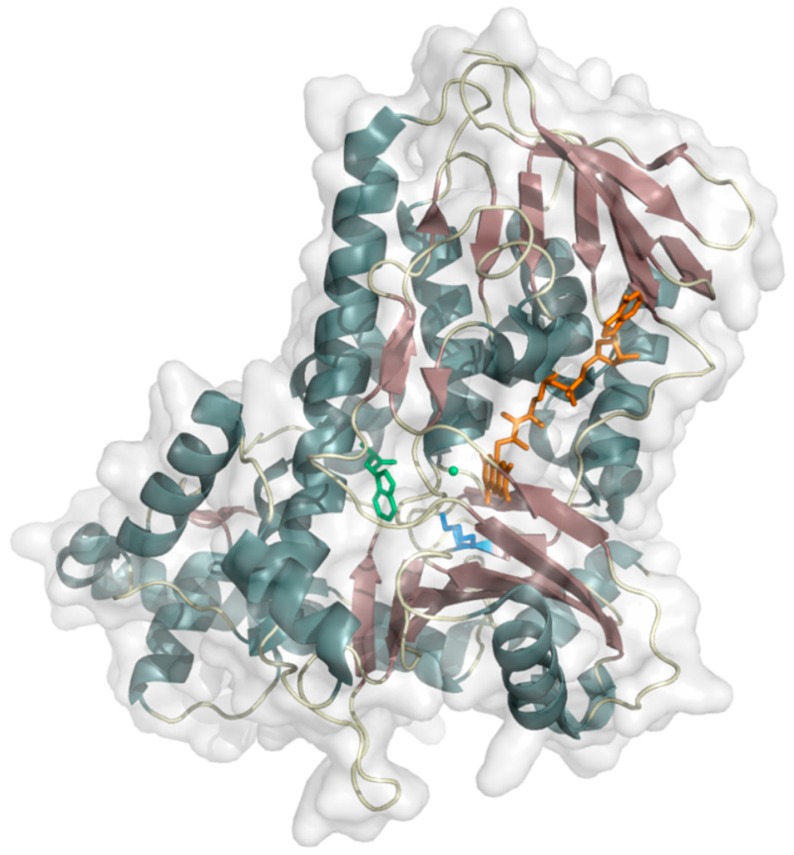
Structure of tryptophan 7-halogenase monomer (PDB ID: 2AQJ). Coloring is according to secondary structure: helices (deep teal), sheets (brown), and loops (pale yellow). The surface is indicated transparently. The FAD cofactor is in orange and L-tryptophan and chloride are in green. Lys79 is indicated in blue [197].

**Biochemistry**. The tryptophan halogenase is a 60-kDa protein that is strictly FAD-dependent. For catalysis, reduced FAD is utilized to reduce oxygen to the flavin C4a-hydroperoxide, which subsequently reacts with a chloride anion to form hypohalous acid (HOCl) and water in the active site [197,198,203]. Tryptophan and FAD are about 10 Å distant from each other within the protein [197]. Thus, HOCl is funneled through the enzyme and where activation occurs via a hydrogen bonding interaction with a lysine residue close to the tryptophan binding site (Figure 9) [218,219]. This adduct is assumed to direct the halogenation of the tryptophan by electrophilic aromatic substitution [219]. In RebH, a chloramine was detected and hypothesized to be the chlorinating agent [220] but might be not strong enough for halogenation [218]. The orientation of the tryptophan in the active site determines the regioselectivity [221] and can be changed by site-directed mutations [221,222,223]. Interestingly, stabilization of the C4a-hydroperoxyflavin is required for the bound substrate in this halogenase [94]. This behaviour is similar to that of one-component FAD-dependent monooxygenases [224] and differs from that of the above mentioned two-component systems. Besides its natural substrate, these halogenases can utilize a variety of tryptamine derivatives, the tricyclic tryptoline, indole and substituted naphthalenes [198,225].

The halogenases require reduced FAD and in many cases flavin reductases are encoded together in a gene cluster with them. For example, the 20-kDa oxidoreductases PrnF and RebF are located in proximity to PrnA and RebH, respectively [217,226]. Yet, in contrast to other two-component systems, it is not clear whether these reductases couple with the halogenases. Direct interaction between the halogenase and the reductase is not necessary for catalysis [227] and it is proposed that these reductases rather supported another oxygenase within the gene cluster [226]. However, RebF is, in analogy to group D and E reductases, strictly NADH-dependent but does not discriminate between FAD and FMN as co-substrates.

**Structure**. Two structures of the tryptophan 7-halogenase were solved with bound FAD and substrate (PDB ID: 2AQJ and 2OA1) (Figure 9). The overall fold of these is similar to that of group A and group E flavoprotein monooxygenases. While binding of the FAD co-substrate is also similar, the tryptophan binds distant from the position of the flavin. The chloride anion binds close to the *re*-side of the isoalloxazine ring of FAD, in agreement with its reaction with the C4a-hydroperoxyflavin [197,228].

#### 2.3.2. Carrier Protein-Bound Substrate Halogenases

**Physiological context**. A second subtype of group F catalyzes the halogenation of a carrier protein (CP)-associated substrate for natural product synthesis and these halogenases are part of non-ribosomal peptide synthesis or polyketide synthesis [192,194]. The first report for halogenation of such a tethered substrate was PltA, which is involved in pyoluteorin biosynthesis [209]. 

**Biochemistry and Structure**. These halogenases can further be distinguished by their action on either pyrrolyl or tyrosyl residues [192]. They require their substrate to be bound to the CP. The CPs guide the growing molecule through the biosynthesis pathway allowing for correct assembly of the final natural product and it is possible that protein–protein interaction between the halogenase and the CP is needed for catalysis [229]. However, the mechanism of these halogenases is not fully understood yet [194]. In particular, there is so far no structure available with bound CP-tethered substrate and therefore the positioning towards the active site remains unclear or can only be estimated by homology modelling [210].

The only characterized flavin reductase, which is encoded in a biosynthesis cluster in proximity of a CP halogenase, is SgCE6 [109,230]. The clusters allow for production of the enediyne antitumor antibiotic C-1027 and contain a group D halogenase [231]. It is also hypothesized that it supplies the hydroxylase as well as the halogenase with reduced FAD [230]. However, there is no evidence for direct interaction between the reductase and the monooxygenases, which is reasonable as it has to act in concert with both [230]. The crystal structure of SgcE6 is similar to other HpaC-like flavin reductases but it is strictly dependent on FAD [109,230].

#### 2.3.3. Biotechnological Relevance of Group F Flavoprotein Monooxygenases

Regioselective halogenated aromatic compounds are used as building block for chemical synthesis as well as pharmaceutical, polymer and agrochemical industry [191,198,203]. A great proportion of drugs and in particular agrochemicals are halogenated as this is often crucial for their biological effect [194,195]. This comprises the type of halogen as well as the position within the molecule what in turn is required for a regioselective, substrate-specific enzymatic halogenation. 

The substrate spectra of group F halogenases includes anilines, indoles, azoles, and pyrroles [191,232]. The halogenases can be integrated into biosynthetic pathways of natural products to generate derivatives of the original compound or non-natural products [195,207,229,233,234,235,236,237]. In particular, the introduction of halogens into natural compounds is a promising strategy to improve products like antibiotics and enhance their therapeutic properties [238,239].

Limitations according to their usability were attributed to their narrow substrate scope, low enzyme production, and stability. However, significant progress in production was achieved by co-expression with chaperones [198]. Their applicability in a biotechnological process was further shown, accompanied by immobilization of the halogenase RebH on a gram scale. The halogenase and the enzymes for cofactor regeneration (reductase PrnF and alcohol dehydrogenase) were co-immobilized as cross-linked enzyme aggregates [240].

Several approaches addressed the improvement of the stability and substrate preference of the halogenases. It was shown that engineering of halogenases can alter the regioselectivity [196,203,222,223,241,242,243] and expand the substrate scope of these enzymes [190,196,203,236,242,243,244]. Similar to group E monooxygenases, it was possible to create functional fusion proteins of halogenase and reductase [245]. In addition, a thermophilic halogenase was identified that is a promising template for further optimizations [246].

## 3. Discussion

Two-component FAD-dependent monooxygenases are versatile enzymes that are involved in many metabolic processes. They are mainly involved in the biosynthesis and degradation of aromatic compounds. They employ flavin:NADH oxidoreductases for generating reduced flavin and in some cases two monooxygenases that are involved in the same enzymatic cascade, accepting the flavin from one reductase [230].

The reductase components show higher sequence identities compared to the monooxygenase components, which might point to a slower evolution [90]. In addition, similar reductases can also be found in other two component enzyme systems [90,226]. This is reasonable, as the pressure towards the oxygenase to convert a different substrate, which is available for the host organism, is likely higher than for the reductase, which delivers the same flavin co-substrate in each case. Nevertheless, some differences are observed in flavin binding, activity, and kinetic mechanisms of the reductases. Except for PheA2, usually no tightly bound FAD is present and thus the FAD is rather a co-substrate than a cofactor. As a result, PheA2 employs a ping-pong mechanism, while the other ones usually follow a sequential mechanism. Most of the reductases are promiscuous towards the flavin that can be reduced. For example, this was reported for PheA2 [69], RebF [217], and HpaC [89]. However, several systems are known, where FAD specificity is not restricted to the monooxygenase component: SgcC/SgcC3/SgcE6 [60,230], NphA1/NphA2 [247,248], TftD/TftC [98], HPAH C2/C1 [80], 4-nitrophenol monooxygenase [249], pyrrole 2-carboxylate monooxygenase [51], and *Ro*StyA/*Ro*StyB [77].

**Figure 10:**
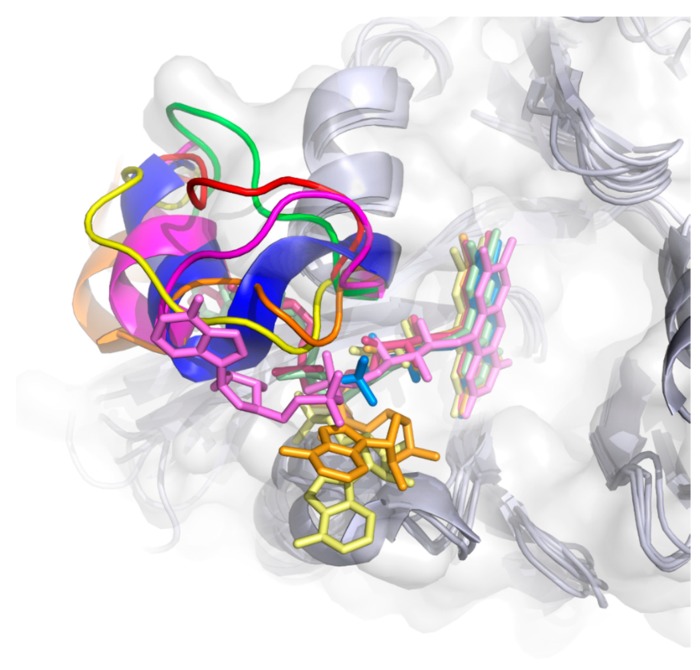
Zoom on the disordered loop region of two-component flavin:NADH reductases. The protein backbone is of the reductases shaded in blue/white. The loop region is illustrated according to PheA2 (PDB ID: 1RZ1, red), HpaC (2ED4, green), HpaC (2D37, blue), SMOB (4F07, yellow), BorF (5CHO, orange), and CobR (4IRA, magenta). Respective flavin ligands are shaded in pale tints.

These differences in flavin substrate specificity are remarkable as the structural similarity between the reductases is high. Two regions in the protein are variable and seem to be responsible for that. First, the *N*-terminus shows high flexibility and differs in length. As this region is assumed to be involved in binding of the nicotinamide and/or a second flavin, the conformation and length of the *N*-terminus might influence the properties of the reductase. Second, a loop region in proximity to the flavin binding site is highly variable in length, orientation and amino acid composition (Figure 10). Structural comparisons indicate that these differences determine the orientation of the adenine moiety of FAD [109]. In some cases, when the loop has an open conformation, that moiety adheres to the protein surface. When the loop closes the respective binding site, the adenine moiety remains solvent-exposed. We see these effects also in proline dehydrogenase from *Thermus thermophilus*, an FAD-dependent oxidoreductase that can also act with FMN [250].

The question arises as to whether there is a physiological reason for that. It is possible that the distinct orientations of the adenine moiety reflect different control mechanisms of FAD reduction and transfer. The monooxygenase and reductase components are usually located in one cluster and also expressed concurrently. Thus, the monooxygenase does not depend on a reduced FAD pool from the cellular metabolism. On the other hand, most flavin reductases do not discriminate between different flavins and might therefore reduce FMN and FAD non-preferentially within the cell. This might cause the accumulation of reactive oxygen species and oxidative stress.

The reduced FAD-transfer step is somewhat of an Achilles heel for the two-component flavin monooxygenase due to the susceptibility of reduced FAD to react with oxygen in aqueous solution with the generation of reactive oxygen species. The two-component monooxygenases employ several approaches to minimize deleterious reactive oxygen species-generating reactions by minimizing the concentration of free flavin in solution. In the thermodynamic cycle of flavin exchange, the reductase component has a high affinity for the oxidized form of FAD, while the monooxygenase preferably binds reduced FAD. This arrangement ensures that upon reduction, the flavin is efficiently sequestered to react productively with the epoxidase. Flavin channelling through a direct protein-protein interaction can further contribute to the efficiency of the FAD-transfer reaction [17,89,152] and the activity of the reductase can influence the rate of the monooxygenase [51,69,73,81]. In those cases, where the reductase does not discriminate between the flavins, it might be possible that the adenine moiety of FAD remains bound within the monooxygenase and only the isoalloxazine system is transferred for reduction within the reductase, allowing for efficient coupling. However, this concept has not been proven so far. The above mentioned strategies for efficient handover of reduced FAD might explain the different binding modes as shown in Figure 10. 

From a biotechnological point of view, it can be advantageous that the monooxygenases can operate independently from the reductase. Flavin regeneration can be accomplished by only one reductase, especially in a cascade reaction where several of these two-component monooxygenases are coupled. Additional flexibility is inferred by the fact that nicotinamide analogues allow for direct reduction of flavins, making the final systems even more simple [39,40,41,42]. The herein mentioned FAD-dependent enzymes catalyze epoxidations, hydroxylations, and halogenations as well as heteroatom oxidations on aromatic substrates in an often regio- and enantioselective manner. Many of them are further able to act on substituted, halogenated, or bulky derivatives of the natural substrate. These reactions are of high interest and hardly accessible by chemical transformations. Exploration and engineering of these enzymes can provide a biotechnological toolbox for the environmental friendly supply of e.g., pharmaceuticals, fine- chemicals, and agrochemicals. 

Natural enzyme cascades that include two FAD-dependent monooxygenases as found in the biosynthesis pathway of the antibiotic C-1027 can serve as a template for artificial production lines [212]. Herein, one reductase SgcE6 is able to generate reduced FAD for both monooxygenases [109,230]. Such bio- or chemobiocatalytic cascades can be used to develop green processes and novel products [167,185,186,251,252,253,254]. In addition, linking of artificial fusion proteins might optimize the processes and simplify the co-substrate/cofactor regeneration [157,188,245].

## 4. Conclusions

Two-component FAD-dependent monooxygenases are able to catalyze reactions that are of high interest for several industrial applications. Several challenges of enzymatic catalysis like limited stability, selectivity, or activity as well as cofactor regeneration certainly apply to these enzymes. Considerable effort has been made in recent years to identify and engineer promising variants. Further attempts focused on the optimization of in vivo and in vitro processes with the aim to use these enzymes at a large scale. Although there is still space for optimization, first large-scale demonstrations showed that these monooxygenases can be a real support and/or alternative to chemical catalysis.

## Figures and Tables

**Table 1:** Representatives of Group D.

Enzyme	EC Number	Reference
Pyrrole 2-carboxylate monooxygenase	1.14.13.130	[51]
4-Nitrocatechol 4-monooxygenase	1.14.13.166	[52]
4-Nitrophenol 4-monooxygenase	1.14.13.167	[52]
2-Aminobenzoate 3-monooxygenase	1.14.14.8	[53]
4-Hydroxyphenylacetate 3-hydroxylase	1.14.14.9	[54]
Phenol 2-hydroxylase	1.14.14.20	[55]
Isobutylamine N1-monooxygenase	1.14.14.30	[49]
2,4,5-Trichlorophenol monooxygenase	-	[56]
2,4,6-Trichlorophenol 4-monooxygenase	-	[57]
4-Chlorophenol monooxygenase	-	[58]
4-Chlorophenol dechlorinase	-	[59]
(*S*)-3-Chloro-*β*-tyrosine hydroxylase	-	[60]
Indosespene cyclase XiaF	-	[48]
3-HSA	-	[61]
Sulfonamide monooxygenase	-	[62]
MeaX monooxygenase	-	[63]
Dibenzothiophene monooxygenase	-	[64]
4-Fluorophenol monooxygenase	-	[65]
Indole 3-acetate monooxygenase	-	[66]
3-Hydroxyphenylacetate 6-hydroxylase	-	[67]

**Table 2:** Representatives of Group E.

Enzyme	EC Number	References
Styrene monooxygenase	1.14.14.11	[119,120,121,122,129,130,131]
Indole monooxygenase	-	[121,123,124,126,127]

**Table 3:** Comparison of critical amino acid residues that are probably involved in nicotinamide binding in PHBH with respective residues of StyA and PqsL [12,151].

Enzyme	PDB ID	1	2	3	4	5	6	7	8
PqsL	2X3N	Q36	R41	I43	G45	I163	A164	R168	G275
SMOA	3IHM	D33	Y39	R43	N46	G144	K145	G149	V274
PHBH	1PBE	R33	Y38	R42	R44	F161	H162	R166	R269

**Table 4:** Representatives of Group F.

Enzyme	EC Number	Reference
**Free substrate halogenases**		
Typtophan 7-halogenase	1.14.19.9	[197,198]
Tryptophan 2-halogenase ^(a)^	-	[199]
Tryptophan 4-halogenase ^(b)^	-	-
Tryptophan 5-halogenase	-	[200]
Tryptophan 6-halogenase	-	[201,202,203,204,205]
Indole-3-halogenase	-	[192]
Fungal halogenases	-	[206,207,208]
**Carrier protein-bound substrate halogenases**		
Pyrrolyl-*S*-PCP halogenases	-	[209,210,211]
(*S*)-*β*-Tyrosyl-*S*-PCP halogenase	-	[212]
Chondrochloren halogenase ^(a)^	-	[213]
Chondramide halogenase ^(a)^	-	[199]
Chloramphenicol halogenase ^(a)^	-	[214]
Tiacumin halogenase ^(a)^	-	-
Monodeoxypyoluteorin *C*3-halogenase ^(a)^	-	[215]
Monodeoxypyoluteorin *N*-halogenase ^(a)^	-	[215]

^(a)^ Physiological role of the halogenase was derived from the chemical structure of the secondary metabolite and the sequence homology with known flavin-dependent halogenases or ^(b)^ solely from the structure of the metabolite.
